# Enzymatic Synthesis of Novel and Highly Soluble Puerarin Glucoside by *Deinococcus geothermalis* Amylosucrase

**DOI:** 10.3390/molecules27134074

**Published:** 2022-06-24

**Authors:** Hsiou-Yu Ding, Tzi-Yuan Wang, Jiumn-Yih Wu, Yu-Li Tsai, Te-Sheng Chang

**Affiliations:** 1 Department of Cosmetic Science, Chia Nan University of Pharmacy and Science, No. 60 Erh-Jen Rd., Sec. 1, Jen-Te District, Tainan 71710, Taiwan; ding8896@gmail.com; 2 Biodiversity Research Center, Academia Sinica, Taipei 11529, Taiwan; tziyuan@gmail.com; 3 Department of Food Science, National Quemoy University, Kinmen County 892, Taiwan; wujy@nqu.edu.tw; 4 Department of Biological Sciences and Technology, National University of Tainan, Tainan 70005, Taiwan; aa0920281529@gmail.com

**Keywords:** amylosucrase, glycosylation, puerarin

## Abstract

Puerarin (daidzein-8-*C*-glucoside) is an isoflavone isolated from several leguminous plants of the genus *Pueraria*. Puerarin possesses several pharmacological properties; however, the poor solubility of puerarin limits its applications. To resolve this poor solubility, *Deinococcus geothermalis* amylosucrase (*Dg*AS) was used to modify puerarin into more soluble derivatives. The results showed that *Dg*AS could biotransform puerarin into a novel compound: puerarin-4′-*O*-*α*-glucoside. The biotransformation reaction was manipulated at different temperatures, pH values, sucrose concentrations, reaction times, and enzyme concentrations. The results showed that the optimal reaction condition was biotransformed by 200 μg/mL *Dg*AS with 20% (*w*/*v*) sucrose at pH 6 and incubated at 40 °C for 48 h, and the optimal production yield was 35.1%. Puerarin-4′-*O*-*α*-glucoside showed 129-fold higher solubility than that of puerarin and, thus, could be further applied for pharmacological use in the future.

## 1. Introduction

Puerarin (daidzein-8-*C*-glucoside) is an isoflavone isolated from several leguminous plants of the genus *Pueraria*. Puerarin possesses several pharmacological properties and was approved for clinical trials in diabetes mellitus (DM) by the Chinese Ministry of Health in 1993 [1]. Recently, puerarin has proved to be a potential anticancer drug [2]. However, its water solubility remains low although puerarin contains a glucosyl residue attached to the parent isoflavone [3]. Thus, its low solubility makes puerarin difficult to apply in food processing and results in poor absorption after oral administration. Only two dosage forms could be found on the market until now: puerarin injections and eye drops [4]. Therefore, improving the solubility of puerarin could strengthen its applications.

Molecular modifications could be achieved by either chemical or enzymatic approaches [5]. However, chemical modifications sometimes involve multiple steps of protection/deprotection to control regioselectivity. Multiple-step reactions usually reduce the final product yield. In contrast, one-step enzymatic modification could promote regio- and enantioselective catalytic activity. For example, glycosylation can significantly improve the solubility of molecules [6]. Glycosylation modulates the solubility, bioavailability, and chemical properties of many natural products, such as flavonoids and steroids [7]. Many novel bioactive flavonoid glycosides have been obtained by enzymatic glycosylation for developing new and potential drugs [8]. 

To develop highly soluble puerarin glycosides, two major forms—*α*-(1→6″)-puerarin and *α*-(1→4″)-puerarin—biotransformed with different enzymes have been previously reported. Li et al., (2004) used the *Bacillus stearothermophilus* maltogenic amylase (*Bs*MA), a glycoside hydrolase family 13 (GH13) (EC 3.2.1.133) enzyme, to glycosylate puerarin with *β*-cyclodextrin (CD) and to produce glucosyl-*α*-(1→6″)-puerarin and maltosyl-*α*-(1→6″)-puerarin, which possessed 14-fold and 168-fold higher aqueous solubility than that of puerarin, respectively [9,10]. Similarly, Li et al., (2011) used the archaeon *Thermofilum pendens* MA (*Tf*MA) to produce the same derivative [11]. Ko et al., (2012) used *Leuconostoc lactis* dextransucrase (GH70; EC 2.4.1.5) to produce glucosyl-*α*-(1→6″)-puerarin and maltosyl-*α*-(1→6″)-puerarin, which possessed 15-fold and 202-fold higher solubility than that of puerarin, respectively [12]. Huang et al., (2020) used another GH13 enzyme, *Bacillus licheniformis* cyclodextrin glucanotransferase (EC 2.4.1.19), to produce glucosyl-*α*-(1→4″)-puerarin, maltosyl-*α*-(1→4″)-puerarin, and maltotriosyl-*α*-(1→4″)-puerarin, which showed 15-fold, 100-fold, and 179-fold higher solubility than that of puerarin, respectively [13]. On the other hand, Wang et al., (2014) and Wu et al., (2013) used *Arthrobacter nicotianae β*-fructosidase (GH32; EC 3.2.1.153) to produce fructosyl-*β*-(2→6″)-puerarin and difructosyl-*β*-(2→6″)-puerarin [14,15], although the authors did not determine the solubility of the two puerarin fructosides. Recently, Nunez-Lopez et al., (2019, 2020) used *Gluconacetobacter diazotrophicus* levansucrase (GH68; EC 2.4.1.10) to produce the same fructosyl-*β*-(2→6″)-puerarin, which showed 23-fold higher solubility than that of puerarin [16,17].

Amylosucrase (AS, EC 2.4.1.4) is another GH13 versatile sucrose-hydrolyzing enzyme [18,19] which uses sucrose as the sole substrate to catalyze the *α*-1,4-glucans derivatives. AS has been proven to glycosylate many flavonoids, such as piceid [20], catechin [21], baicalein [22], isoquercetrin [23,24], rutin [25], phloretin [26], taxifolin, aseculetin, and luteolin [27]. For example, previous studies used *Deinococcus geothermalis* amylosucrase (*Dg*AS) to glycosylate soyisoflavone 8-hydroxydaidzein (8-OHDe) and 8-OHDe-7-*O*-glucoside into highly soluble and stable 8-OHDe glucoside and diglucosides [28,29]. Because the chemical structure of puerarin is similar to that of 8-OHDe-7-*O*-glucoside, we expect that puerarin could be glycosylated by *Dg*AS to produce more soluble puerarin analogs. Thus, *Dg*AS with sucrose was used to modify puerarin, and the produced puerarin analog was purified, chemically identified, and characterized in terms of its solubility.

## 2. Results and Discussion

### 2.1. Biotransformation of Puerarin by DgAS

Puerarin was biotransformed by *Dg*AS to modify puerarin. The biotransformation products were analyzed by HPLC. The results showed that *Dg*AS could biotransform puerarin into a major product, compound (**1**) (Figure S1, see Supplementary Materials). Fifteen point six percent of puerarin was converted to compound (**1**) by *Dg*AS under the original testing conditions.

**Figure 1:**
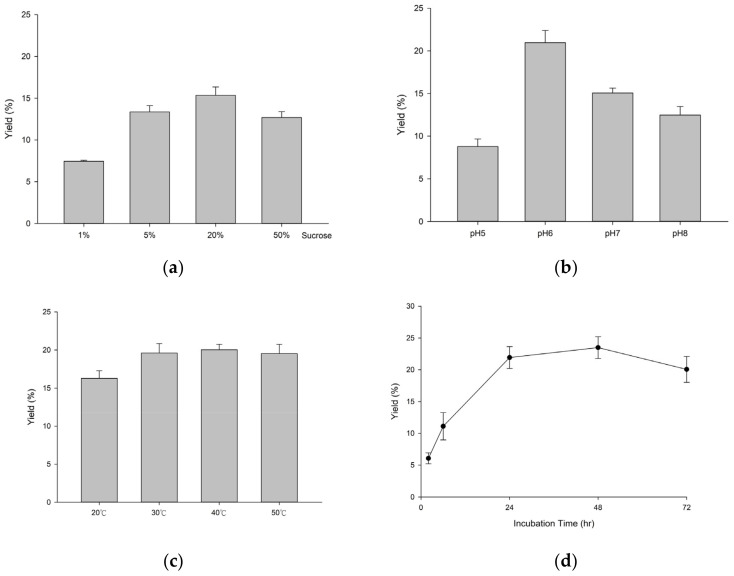
Effects of sucrose concentration (**a**), pH (**b**), temperature (**c**), and time (**d**) on the production of compound (**1**) from biotransformation of puerarin by *Dg*AS. The standard reaction condition was 1 mg/mL of puerarin, 25 μg/mL of *Dg*AS, 50% (*w*/*v*) sucrose, and 50 mM of PB (pH 7) at 40 °C for 24 h. To determine suitable reaction conditions, different sucrose concentrations (*w*/*v*), pH values, temperatures, and reaction times were tested. After the reaction, the reaction product was analyzed by HPLC, and the yield of compound (**1**) was calculated by dividing the HPLC area of compound (**1**) by that of the sum of compound (**1**) and the residual puerarin in the HPLC analysis. The detailed reaction conditions and the HPLC procedure are described in the Materials and Methods section.

**Figure 2:**
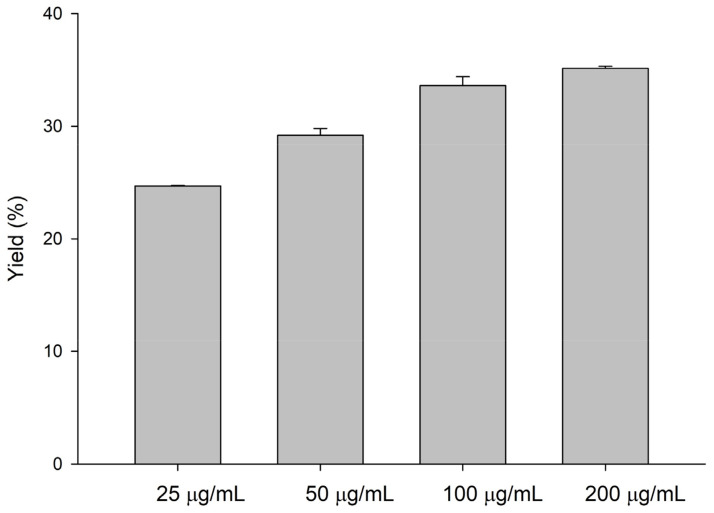
Effects of *Dg*AS concentration on the yields of compound (**1**) biotransformed from puerarin by *Dg*AS. The reaction condition was 1 mg/mL of puerarin, 25 to 200 μg/mL of *Dg*AS, 20% (*w*/*v*) sucrose, and 50 mM of PB (pH 6) at 40 °C for 48 h. After the reaction, the product was analyzed by HPLC, and the yield of compound (**1**) was estimated by dividing the HPLC area of compound (**1**) by that of the sum of compound (**1**) and the residual puerarin. The detailed reaction conditions and the HPLC procedure are described in Section 3.3.

To improve the efficiency of the biotransformation by *Dg*AS, the reaction conditions were optimized with different sucrose concentrations, temperatures, pH values, and times. The results showed that the optimal reaction conditions were 20% (*w*/*v*) sucrose and pH 6 at 40 °C for 48 h (Figure 1). Under the optimal reaction conditions, the yield of compound (**1**) from the biotransformation of puerarin by *Dg*AS was increased to 24.1%. To improve the production yield in advance, the *Dg*AS enzyme concentration was increased from 25 μg/mL to 200 μg/mL in reactions. The results showed that the highest yield of compound (**1**) can reach 35.1% when *Dg*AS was increased to 200 μg/mL (Figure 2).

### 2.2. Identification of the Biotransformation Product

**Figure 3:**
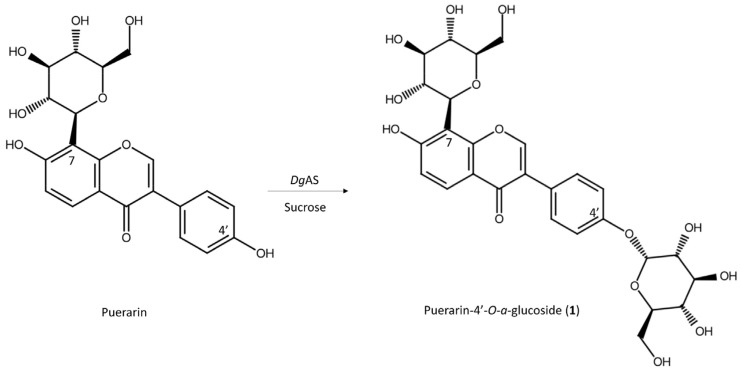
The biotransformation process of puerarin by *Dg*AS.

To reveal the chemical structures of compound (**1**), the biotransformation was scaled up to 40 mL. The biotransformation product was purified by preparative HPLC. The chemical structure of the purified compound was then resolved using mass and nucleic magnetic resonance (NMR) spectral analyses. The molecular formula of compound (**1**) was established as C_27_H_30_O_14_ by the electrospray ionization mass spectrometry (ESI-MS) at *m*/*z* 577.3 [M-H]^−^, indicating the molecular weight of 578 and the presence of a glucoside to puerarin (Figure S2). The functional groups of compound (**1**) were analyzed by infrared (IR) spectroscopy. The results revealed significant absorption at 3273 cm^−^^1^, representing the typical hydroxyl groups, and another at 1623.9 cm^−^^1^, representing the typical carbonyl groups in compound (**1**) (Figure S3). The compound (**1**) characteristic ^1^H and ^13^C NMR sugar signals have been assigned to *C*-glucosyl and *O*-glucosyl moieties by 1D and 2D NMR experiments. The ^1^H spectrum of compound (**1**) in DMSO-d_6_ showed one singlet at 8.42 ppm; eight doublets at 4.81, 5.42, 6.99, 7.13, 7.13, 7.52, 7.52, and 7.94 ppm; and a complex 10-spin system between 3.0 and 5.0 ppm. An analysis of this second-order system revealed coupling constants typical of two glucose moieties. The compound (**1**) glycosidic linkage of the *C*-glucosyl moiety on puerarin C-8 has been revealed by the presence of heteronuclear multiple bond connectivity (HMBC) correlations between C-8/H-1″ (112.7/4.81 ppm) and anomeric proton H-1″ at 4.81 (d, J = 9.1 Hz), indicating a C-*β*-configuration of puerarin, supported by data from previous literature [30]. The puerarin *O*-glucosyl moiety was a doublet signal at H-1‴ (5.42 ppm, d, J = 3.5 Hz), with the corresponding carbon atom at C-1‴ (98.0 ppm) assigned to the anomeric proton, and indicated an *O*-*α*-configuration by heteronuclear single quantum coherence (HSQC), which is in the *O*-*α*-configuration, and the H-1‴ (*δ* = 5.42 ppm) of puerarin, as well as the HMBC cross signals H-1‴/C-4′ (5.42/156.9 ppm). The significant downfield shift in the ^13^C signal of C-4′ indicated the connection of the second glucosyl moiety. The NMR signals were fully identified, as shown in Table S1. Compound (**1**) was thus confirmed to be puerarin-4′-*O*-*α*-glucoside (Table S1 and Figures S4–S10). Figure 3 illustrates the biotransformation process of puerarin by *Dg*AS.

Previously studied GH enzymes catalyzed two major glycosylations—*α*-(1→6″)-puerarin and *α*-(1→4″)-puerarin—on the *C*-glucoside residue of puerarin [9,10,11,12,13,14,15,16,17]. In contrast, *Dg*AS preferred catalyzing glycosylation on the 4′-hydroxyl group of puerarin and produced a novel derivative. Our previous study also showed that *Dg*AS catalyzed glycosylation on the 4′-hydroxyl group of 8-OHDe-7-*O*-glucoside and produced 8-OHDe-7,4′-*O*-diglucoside [29]. Both studies suggest that *Dg*AS is a good enzyme for producing isoflavones-4′-glucoside.

### 2.3. Aqueous Solubility of Puerarin and Its Derivatives

The solubility of puerarin and its derivatives was determined by HPLC analysis. The results showed that puerarin-4′-*O*-*α*-glucoside had a solubility 129 folds higher than that of puerarin (Table 1). The well soluble puerarin-4′-*O*-*α*-glucoside could present an alternative application to the industry.

It is known that the more sugars are attached, the higher the solubility of the modified molecules. Table 2 summarizes various puerarin glycosides produced by different GH enzymes. All reported puerarin-monoglycosides linked to the C8-glucoside of puerarin possessed glycosyl attached to one site (C8). Most puerarin-monoglycosides possessed 14-fold to 23-fold higher aqueous solubility than that of puerarin. Furthermore, puerarin-diglycosides or puerarin-triglycosides possessed over 100-fold higher aqueous solubility than that of puerarin. On the other hand, the solubility of puerarin-4′-*O*-*α*-glucoside (puerarin-monoglycoside) was 129 folds higher than that of puerarin. A possible reason for such higher aqueous solubility might be the mono-sugar linked to the two glucosyl sites (C8 and C4′) of puerarin-4′-*O*-*α*-glucoside. Higher solubility of a similar linkage was also found in the 8-OHDe-7,4′-*O*-*α*-diglucoside, which was linked to the two glucosyl sites (C7 and C4′); it was seven folds higher than that of daidzin-4″-*O*-*α*-glucoside [29]. This study revealed that glycosylation at the 4′-hydroxyl group on the isoflavone skeleton would yield better solubility than glycosylation at the hydroxyl groups on the C-glucoside of puerarin. This study highlighted a novel and unique action of *Dg*AS on the 4′-glycosylation of isoflavones.

Previous studies revealed that *O*-glycosylated flavonoids (vitexin, isovitexin, and isoorientin) could be deglycosylated to recover the bioactivities of the parental flavonoids via in vitro fecal fermentation [31] or in vivo intestinal microbes [32]. In contrast, it has been reported that C-glucosides (e.g., puerarin) are more resistant to acidic and enzymatic hydrolysis [31]. Thus, the novel puerarin glucoside (*O*-glucoside) might also be in vivo digested to the parental puerarin as previous studies has shown [31,32]. Nevertheless, we expect that the high solubility of puerarin glucosides is an advantage for clinical therapy, in which the puerarin glucosides could be absorbed more easily and deglycosylated into the human body with a wider range of pharmaceutical dosages. Further clinical experiments are needed to confirm the bioactivities in the future.

## 3. Materials and Methods

### 3.1. Microorganism and Chemicals

Puerarin was purchased from Baoji Herbest Bio-Tech (Xi-An, Shaanxi, China). Recombinant *Dg*AS was obtained from our previous studies [28,29]. One unit of *Dg*AS activity was defined as the amount of the enzyme that hydrolyzed sucrose into 1 μmol of fructose per minute. The specific sucrose hydrolysis activity of the purified recombinant *Dg*AS was determined to be 6.6 U/mg. The other reagents and solvents used were commercially available. 

### 3.2. Biotransformation Using DgAS

The reaction mixture (0.1 mL) comprised 25 μg/mL of *Dg*AS, 1 mg/mL of the tested substrate compound (diluted from a stock of 20 mg/mL in DMSO), 50% (*w*/*v*) sucrose, and 50 mM of phosphate buffer (PB) at pH 7 and was incubated at 40 °C for 24 h. The reaction was stopped by adding an equal volume of methanol and was analyzed using high-performance liquid chromatography (HPLC). To optimize the reaction conditions, different temperatures, pH values, sucrose concentrations, and reaction times were used. The buffers used were 50 mM of acetate buffer (pH 5), PB (pH 6 and 7), and Tris buffer (pH 8). 

### 3.3. HPLC Analysis

HPLC was performed with the Agilent 1100 series HPLC system (Santa Clara, CA, USA) equipped with a gradient pump (Waters 600, Waters, Milford, MA, USA). The stationary phase was a C18 column (5 μm, 4.6 i.d. × 250 mm; Sharpsil H-C18, Sharpsil, Beijing, China), and the mobile phase was 1% acetic acid in water (A) and methanol (B). The elution condition was a linear gradient from 0 min with 40% B to 20 min with 70% B, an isocratic elution from 20 min to 25 min with 70% B, a linear gradient from 25 min with 70% B to 28 min with 40% B, and an isocratic elution from 28 min to 35 min with 40% B. All eluants were eluted at a flow rate of 1 mL/min. The sample volume was 10 μL. The detection condition was set at 254 nm.

### 3.4. Purification and Identification of the Biotransformation Metabolite

The purification process followed a previously described method [33]. To purify compound (**1**), the biotransformation reaction was scaled up to 40 mL (1 mL per tube), and the 40-vial reactions were incubated with 180 rpm of shaking at 40 °C for 24 h. After the reaction, compound (**1**) was purified by a preparative YoungLin HPLC system. The fraction with the compound (**1**) was collected, condensed under a vacuum, and then dehydrated by freeze drying. In total, five batches of the 40 mL reactions were purified for 60.1 mg of compound (**1**). The production yield was 60.1 mg/277.8 mg = 21.6%, which is the purified product (60.1 mg) divided by that theoretical value of 100% conversion rate [(40 mL × 1 mg/mL puerarin)/(416 molecular weight of puerarin) × [578 molecular weight of compound (**1**)] × 5 batches] = 277.8 mg]. The structures of the compound were confirmed with NMR and mass spectral analyses. Mass analyses were performed using the Finnigan LCQ Duo mass spectrometer (ThermoQuest Corp., San Jose, CA, USA) with electrospray ionization (ESI). ^1^H- and ^13^C-NMR, distortionless enhancement by polarization transfer (DEPT), heteronuclear single quantum coherence (HSQC), heteronuclear multiple bond connectivity (HMBC), correlation spectroscopy (COSY), and nuclear Overhauser effect spectroscopy (NOESY) spectra were recorded on a Bruker AV-700 NMR spectrometer at ambient temperature. Standard pulse sequences and parameters were used for the NMR experiments, and all chemical shifts were reported in parts per million (ppm, *δ*).

Puerarin-4′-*O*-*α*-glucoside (**1**): light gray powder; ESI/MS *m*/*z*: 577.3 [M-H]^−^, 457.3, 439.1, 429.3, 414.1, 309.1, 295.2, 294.2, 266.0; IR (HBr): *ν*_max_ = 3273 (OH), 1623.9 (C = O), 1507.4 (C – C), 1010.2 (C – O) cm^−1^; ^1^H-NMR (DMSO-*d_6_*, 700 MHz) H*δ*: 3.13 (2H, m, H-4″, 4‴), 3.24 (1H, m, H-5″), 3.26 (1H, t, *J* = 9.1 Hz, H-3″), 3.35 (1H, m, H-2‴), 3.43 (1H, m, H-6″a), 3.47 (1H, m, H-6‴a), 3.48 (1H, m, H-5‴), 3.64 (1H, m, H-3‴), 3.57 (1H, m, H-6‴b), 3.72 (1H, m, H-6″b), 4.03 (1H, t, *J* = 9.1 Hz, H-2″), 4.81 (1H, d, *J* = 9.1 Hz, H-1″), 5.42 (1H, *J* = 3.5 Hz, H-1‴), 6.99 (1H, d, *J* = 8.8 Hz, H-6), 7.13 (2H, d, *J* = 8.8 Hz, H-3′, 5′), 7.52 (2H, d, *J* = 8.8 Hz, H-2′, 6′), 7.94 1H, d, *J* = 8.8 Hz, H-5), 8.42 (1H, s, H-2). ^13^C-NMR (DMSO-*d_6_*, 175 MHz) C*δ*: 60.7 (C-6‴), 61.4 (C-6″), 69.9 (C-4″, C-4‴), 70.7 (C-2″), 71.6 (C-2‴), 73.1 (C-3‴), 73.4 (C-1″), 73.8 (C-5‴), 78.8 (C-3″), 81,9 (C-5″), 98.0 (C-1‴), 112.7 (C-8), 116.6 (C-4a, 6, 3′, 5′), 122.7 (C-3), 125.6 (C-1′), 126.6 (C-5), 129.9 (C-2′, 6′), 153.1 (C-2), 156.9 (C-8a, 4′), 161.4 (C-7), 174.8 (C-4).

### 3.5. Determination of Solubility

The aqueous solubility of puerarin and its glucoside were determined according to a previous method with slight modification [34]. Twenty milligrams of the tested compound were resuspended in 50 μL of double-deionized H_2_O with 180 rpm of shaking at 25 °C for 1 h. The mixture was then centrifuged at 10,000× *g* at 25 °C for 30 min. The supernatant was filtrated with 0.2 μm of nylon membrane for HPLC analysis. For HPLC analysis, 10 μL of the puerarin filtrate or 2 μL of the puerarin-4′-*O*-*α*-glucoside filtrate were 100-fold or 500-fold diluted with 50% methanol, respectively. The concentrations of the tested compounds were determined with calibration curves of authentic samples. The final concentrations of the standard solutions were prepared with 4, 8, 12, 16, or 20 mg/L of puerarin (from 20 mg/mL of stock in DMSO) or with 100, 200, 300, or 400 mg/L of puerarin-4′-*O*-*α*-glucoside (from 20 mg/mL stock in DMSO) in 50% methanol.

## 4. Conclusions

Puerarin possesses important pharmacology activities for clinical usage. However, the poor solubility property of puerarin limits its dosage. In the present study, puerarin could be glycosylated by *Dg*AS to produce a new compound, puerarin-4′-*O*-*α*-glucoside. Puerarin-4′-*O*-*α*-glucoside showed 129-fold higher solubility than that of puerarin. Such higher solubility could be further applied for pharmacological therapy in the future.

## Figures and Tables

**Table 1:** Aqueous solubility of puerarin and its glucoside.

Compound	Aqueous Solubility (mg/L)	Fold ^1^
Puerarin	2.02 × 10^3^ ± 3.37 × 10^2^	1.0
Puerarin-4′-*O*-*α*-glucoside (**1**)	2.60 × 10^5^ ± 2.86 × 10^3^	128.7

^1^ The fold of aqueous solubility of puerarin glucoside derivatives is expressed relative to that of puerarin, normalized to 1.

**Table 2:** Aqueous solubility of puerarin glycosides reported in the literature.

Puerarin Glycoside	Catalyzed Enzymes	Sugar Donor	Added Sugar	Relative Solubility ^1^	Reference
Puerarin	-	-	0	1	[9,12,13,16], this study
Puerarin-4′-*α*-glucoside	Amylosucrase (*Dg*AS)	Sucrose	1	129	This study
Glucosyl-*α*-(1→6′′)-puerarin	Maltogenic amylase (*Bs*MA); Dextransucrase (*Ll*DexT)	Maltotriose	1	14–15	[9,12]
Maltosyl-*α*-(1→6′′)-puerarin		Sucrose	2	168–202	[9,12]
Glucosyl-*α*-(1→4′′)-puerarin	Glucanotransferase (*Bl*CGT) Glucanotransferase (*Bl*CGT) Glucanotransferase (*Bl*CGT)	Cyclodextrin	1	15	[13]
Maltosyl-*α*-(1→4′′)-puerarin		Cyclodextrin	2	100	[13]
Maltotriosyl-*α*-(1→4′′)-puerarin		Cyclodextrin	3	179	[13]
Fructosyl-*β*-(2→6′′)-puerarin	Levansucrase (*Ls*dA)	Sucrose	1	23	[16]

^1^ Relative solubility was expressed relative to the solubility of puerarin normalized to 1.

## Data Availability

Data is contained within Supplementary Materials.

## Supplementary Materials

The following supporting information can be downloaded at https://www.mdpi.com/article/10.3390/molecules27134074/s1. Table S1: ^1^H and ^13^C NMR assignments in DMSO-*d_6_* at 700 and 175 MHz for compounds (**1**); Figure S1: High-performance liquid chromatography (HPLC) analysis of the biotransformation products of puerarin using *Deinococcus geothermalis* amylosucrase (*Dg*AS); Figure S2: The mass-mass analysis of puerarin-4′-*O*-*α*-glucoside (**1**) at the negative mode; Figure S3: The infrared (IR) analysis of puerarin-4′-*O*-*α*-glucoside (**1**); Figure S4: 1D NMR spectrum (^1^H-NMR, 700 MHz, DMSO-*d_6_*) of the puerarin-4′-*O*-*α*-glucoside (**1**); Figure S5: 1D NMR spectrum (^13^C-NMR, 175 MHz, DMSO- *d_6_*) of the puerarin-4′-*O*-*α*-glucoside (**1**); Figure S6: 1D NMR spectrum (DEPT-135, 175 MHz, DMSO-*d_6_*) of the puerarin-4′-*O*-*α*-glucoside (**1**); Figure S7: 2D NMR spectrum (^1^H-^13^C HSQC, 700 MHz, DMSO-*d_6_*) of the puerarin-4′-*O-α*-glucoside (**1**); Figure S8: 2D NMR spectrum (^1^H-^13^C HMBC, 700 MHz, DMSO-*d_6_*) of the puerarin-4′-*O*-*α*-glucoside (**1**); Figure S9: 2D NMR spectrum (^1^H-^1^H COSY, 700 MHz, DMSO-*d_6_*) of the puerarin-4′-*O*-*α*-glucoside (**1**); Figure S10: 2D NMR spectrum (^1^H-^1^H NOESY, 700 MHz, DMSO-*d_6_*) of the puerarin-4′-*O*-*α*-glucoside (**1**).

## Sample Availability

Samples of compound (**1**) are available from the authors.
