# Supplementary material for: Enzymatic Synthesis of Novel and Highly Soluble Puerarin Glucoside by Deinococcus geothermalis Amylosucrase
Source: Molecules. 2022 Jun 24;27(13):4074. doi: 10.3390/molecules27134074 (PMC9268652; doi:10.3390/molecules27134074)
Supplement: Supplementary file 1 [file molecules-27-04074-s001.zip › molecules-1747281-supplementary.pdf]

Supplementary Materials: Enzymatic Synthesis of Novel and Highly-Soluble Puerarin Glucoside by *Deinococcus geothermalis* Amylosucrase

Hsiou-Yu Ding, [1,+] Tzi-Yuan Wang, [2,+] Jiumn-Yih Wu, [3,+] Yu-Li Tsai [4] and Te-Sheng Chang, [4,*]

[1] Department of Cosmetic Science, Chia Nan University of Pharmacy and Science, No. 60, Sec. 1, Erh-Jen Rd., Jen-Te District, Tainan 717, Taiwan

[2] Biodiversity Research Center, Academia Sinica, Taipei 115, Taiwan

[3] Department of Food Science, National Quemoy University, Kinmen County 892, Taiwan

[4] Department of Biological Sciences and Technology, National University of Tainan, Tainan 700, Taiwan

[*] Correspondence: mozyme2001@gmail.com; Tel.: +886-6-2602137; Fax: +886-6-2606153

[+] The authors contributed equally

Contents

Table S1. ^{1}H and ^{13}C NMR assignments in DMSO-d_6 at 700 and 175 MHz for compounds (**1)**.

Figure S1. High-performance liquid chromatography (HPLC) analysis of the biotransformation products of puerarin using *Deinococcus geothermalis* amylosucrase (*Dg*AS).

Figure S2. The mass-mass analysis of puerarin-4'-*O*-α-glucoside (**1**) at the negative mode.

Figure 3. The infrared (IR) analysis of puerarin-4'-*O*-α-glucoside (**1**).

Figure S4. 1D NMR spectrum (^{1}H-NMR, 700 MHz, DMSO-d_6) of the puerarin-4'-*O*-α-glucoside (**1**).

Figure S5. 1D NMR spectrum (^{13}C-NMR, 175 MHz, DMSO- d_6) of the puerarin-4'-*O*-α-glucoside (**1**).

Figure S6. 1D NMR spectrum (DEPT-135, 175 MHz, DMSO-d_6) of the puerarin-4'-*O*-α-glucoside (**1**).

Figure S7. 2D NMR spectrum (^{1}H-^{13}C HSQC, 700 MHz, DMSO-d_6) of the puerarin-4'-*O*-α-glucoside (**1**).

Figure S8. 2D NMR spectrum (^{1}H-^{13}C HMBC, 700 MHz, DMSO-d_6) of the puerarin-4'-*O*-α-glucoside (**1**).

Figure S9. 2D NMR spectrum (^{1}H-^{1}H COSY, 700 MHz, DMSO-d_6) of the puerarin-4'-*O*-α-glucoside (**1**).

Figure S10. 2D NMR spectrum (^{1}H-^{1}H NOESY, 700 MHz, DMSO-d_6) of the puerarin-4'-*O*-α-glucoside (**1**).

Table S1. ^{1}H and ^{13}C NMR assignments in DMSO-d_6 at 700 and 175 MHz for compounds (**1**).

position	δ_C	δ_H (J in HZ)
2	153.1	8.42, s
3	122.7	
4	174.8	
4a	116.6	
5	126.6	7.94, d, J=8.8 Hz
6	116.6	6.99, d, J=8.8 Hz
7	161.4	
8	112.7	
8a	156.9	
1'	125.6	
2'	129.9	7.52, d, J=8.8 Hz
3'	116.6	7.13, d, J=8.8 Hz
4'	156.9	
5'	116.6	7.13, d, J=8.8 Hz
6'	129.9	7.52, d, J=8.8 Hz
1''	73.4	4.81, d, J=9.1 Hz
2''	70.7	4.03, t, J=9.1 Hz
3''	78.8	3.26, t, J=9.1 Hz
4''	69.9	3.13, m
5''	81.9	3.24, m
6''	61.4	3.43, m
		3.72, m
1'''	98.0	5.42, d, J=3.5 Hz
2'''	71.6	3.35, m
3'''	73.1	3.64, m
4'''	69.9	3.13, m
5'''	73.8	3.48, m
6'''	60.7	3.47, m
		3.57, m

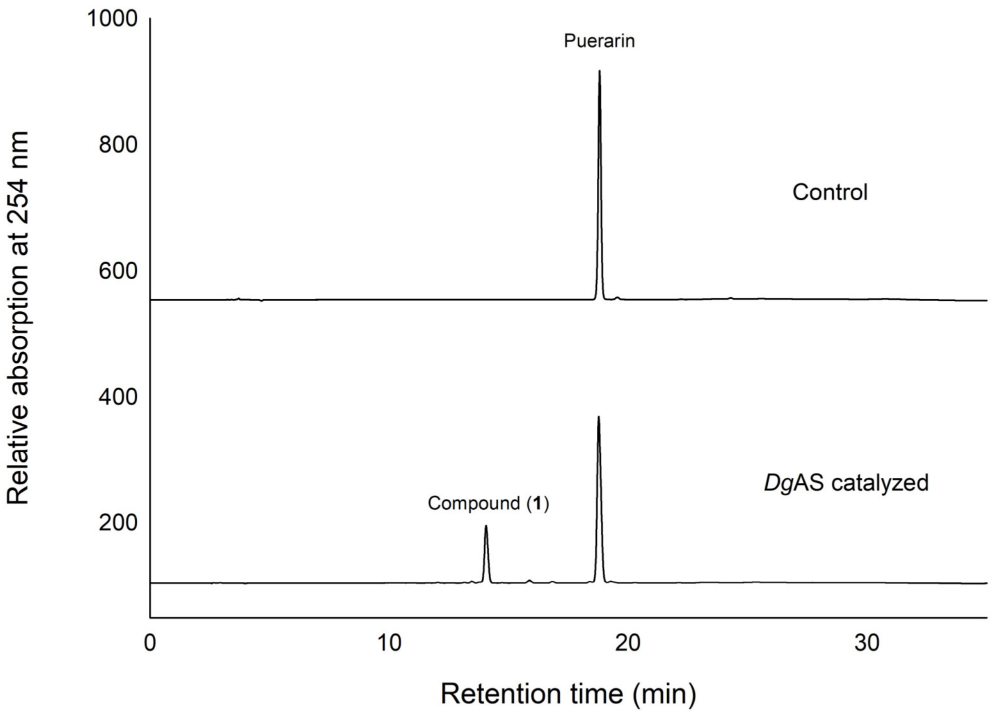

Figure S1. High-performance liquid chromatography (HPLC) analysis of the biotransformation products of puerarin using *Deinococcus geothermalis* amylosucrase (*Dg*AS). The *Dg*AS biotransformation mixture containing 25 ◎g/mL of purified recombinant *Dg*AS enzymes, 1 mg/mL of puerarin, 50% (w/v) sucrose, and 50 mM of phosphate buffer at pH 7 was incubated at 40°C for 24 h. After incubation, the biotransformation products were analyzed by HPLC. The HPLC operation procedure is described in the Materials and methods section.

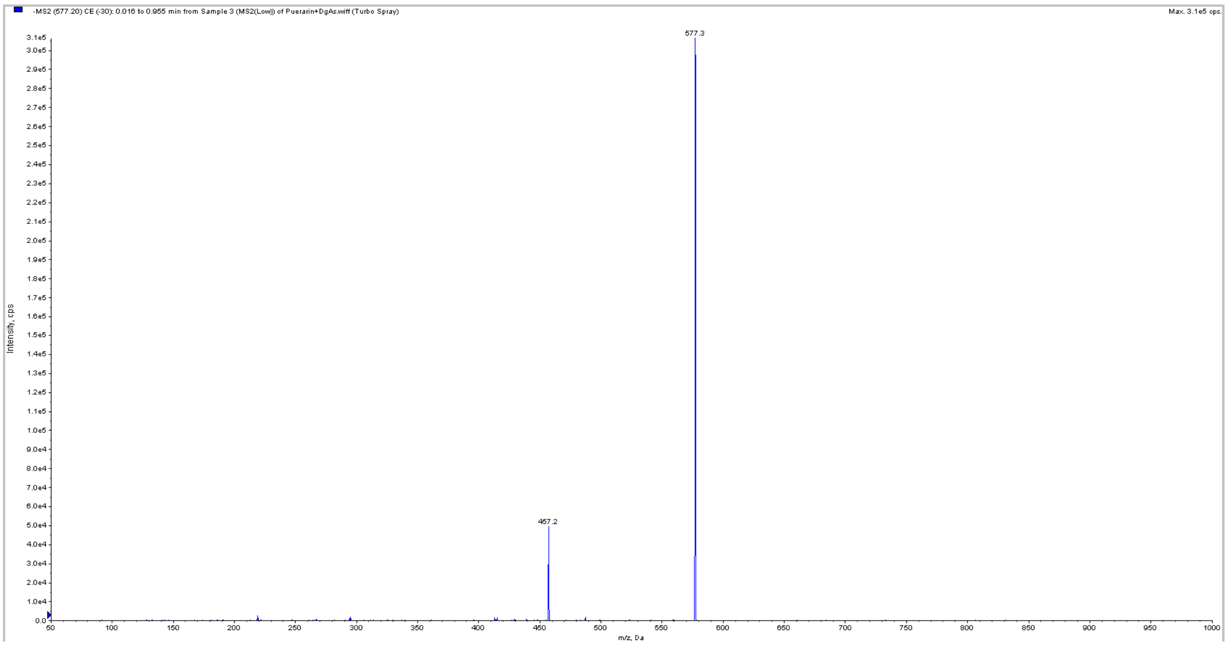

Figure S2. The mass-mass analysis of puerarin-4'-*O*-β-glucoside (**1**) at the negative mode. A significant signal at *m*/*z* 577.3 showed the corresponding *m*/*z* signal of molecular weight 416 of puerarin-4'-*O*-β-glucoside (**1**) (416+180-18-1) at the negative mode [puerarin -G - H]$^{-}$.

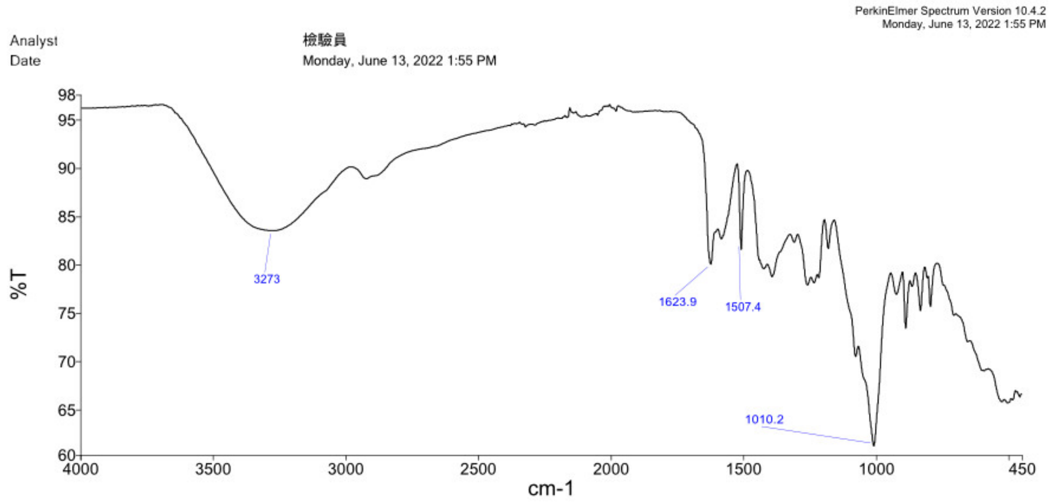

Sample Name	Description	Quality Checks
Pue+G	Sample 160 By FTIR Date Monday, June 13 2022	The Quality Checks give rise to a Weak Bands warning for the sample.

Figure 3. The infrared (IR) analysis of puerarin-4'-*O*-β-glucoside (**1**).

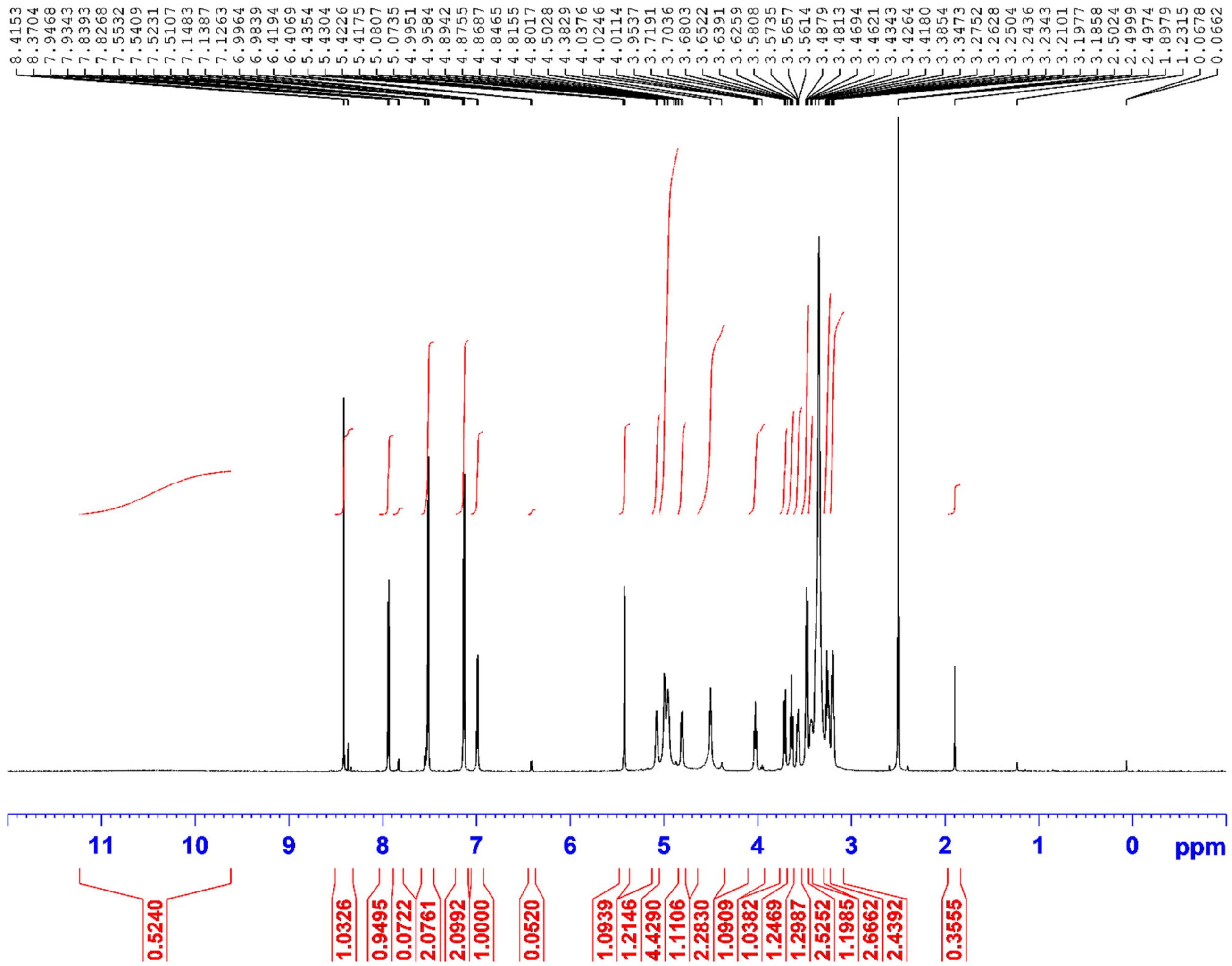

Figure S4. 1D NMR spectrum (^{1}H-NMR, 700 MHz, DMSO-d_6) of the puerarin-4'-*O*-β-glucoside (**1**).

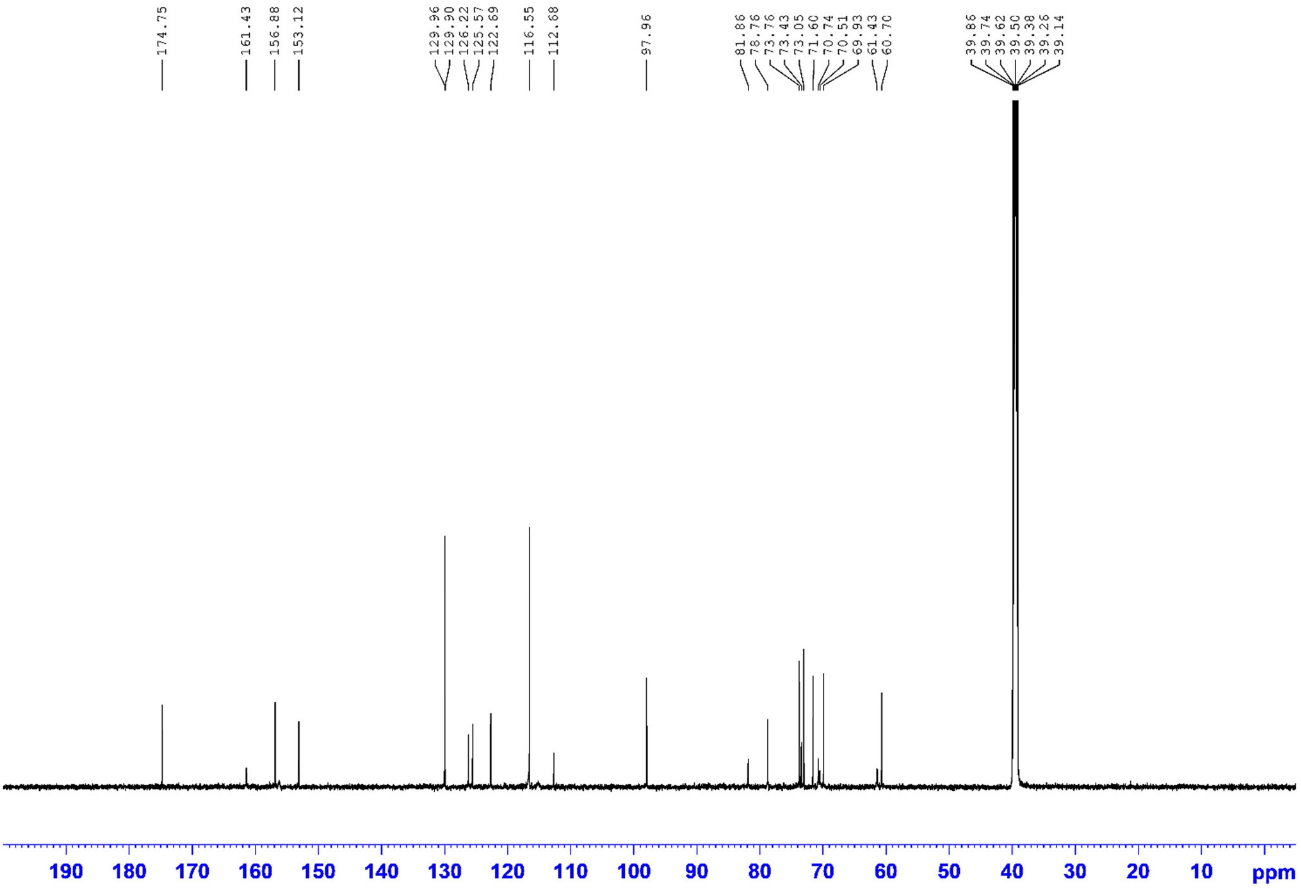

Figure S5. 1D NMR spectrum (^{13}C-NMR, 175 MHz, DMSO- d_6) of the puerarin-4'-*O*-β-glucoside (**1**).

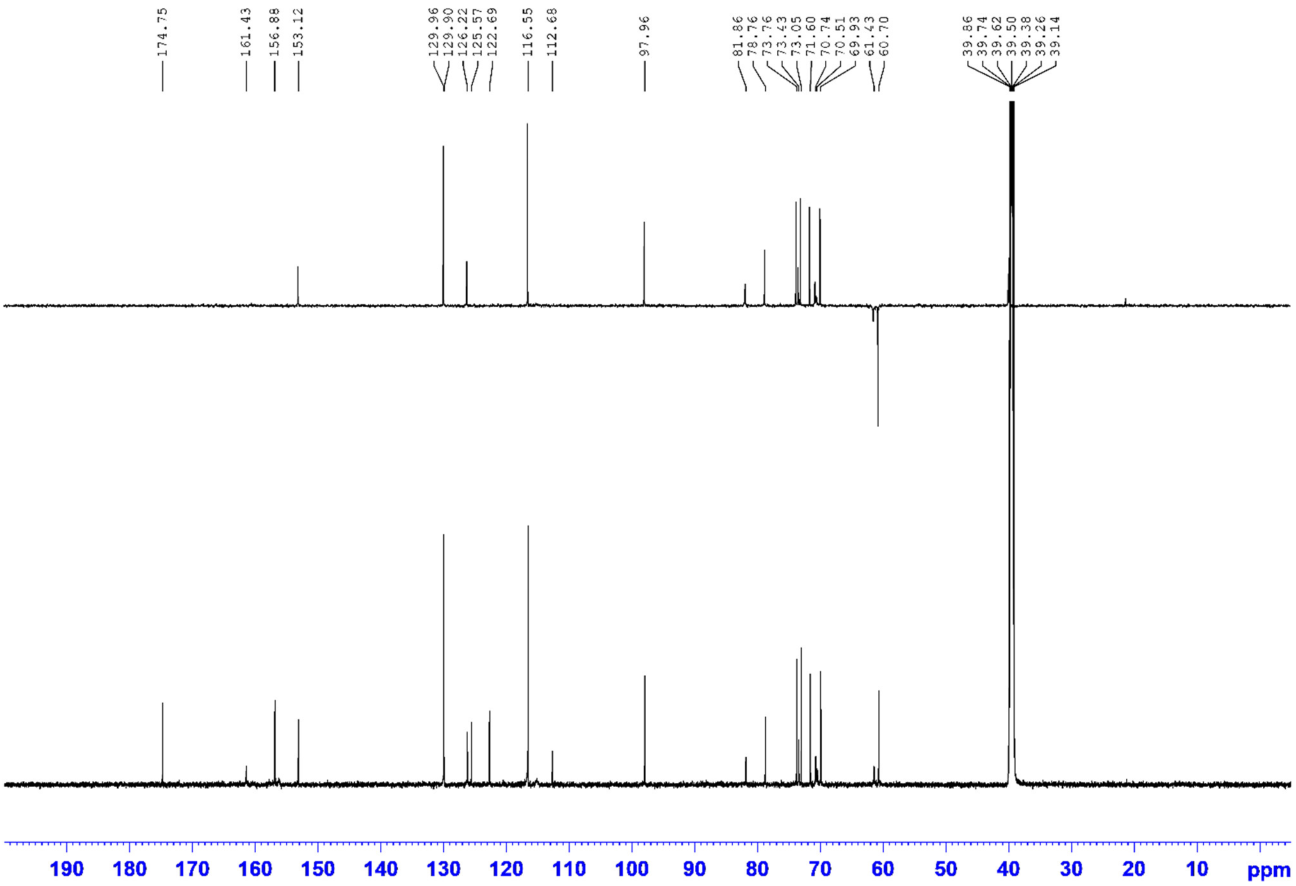

Figure S6. 1D NMR spectrum (DEPT-135, 175 MHz, DMSO-d_6) of the puerarin-4'-*O*-β-glucoside (**1**).

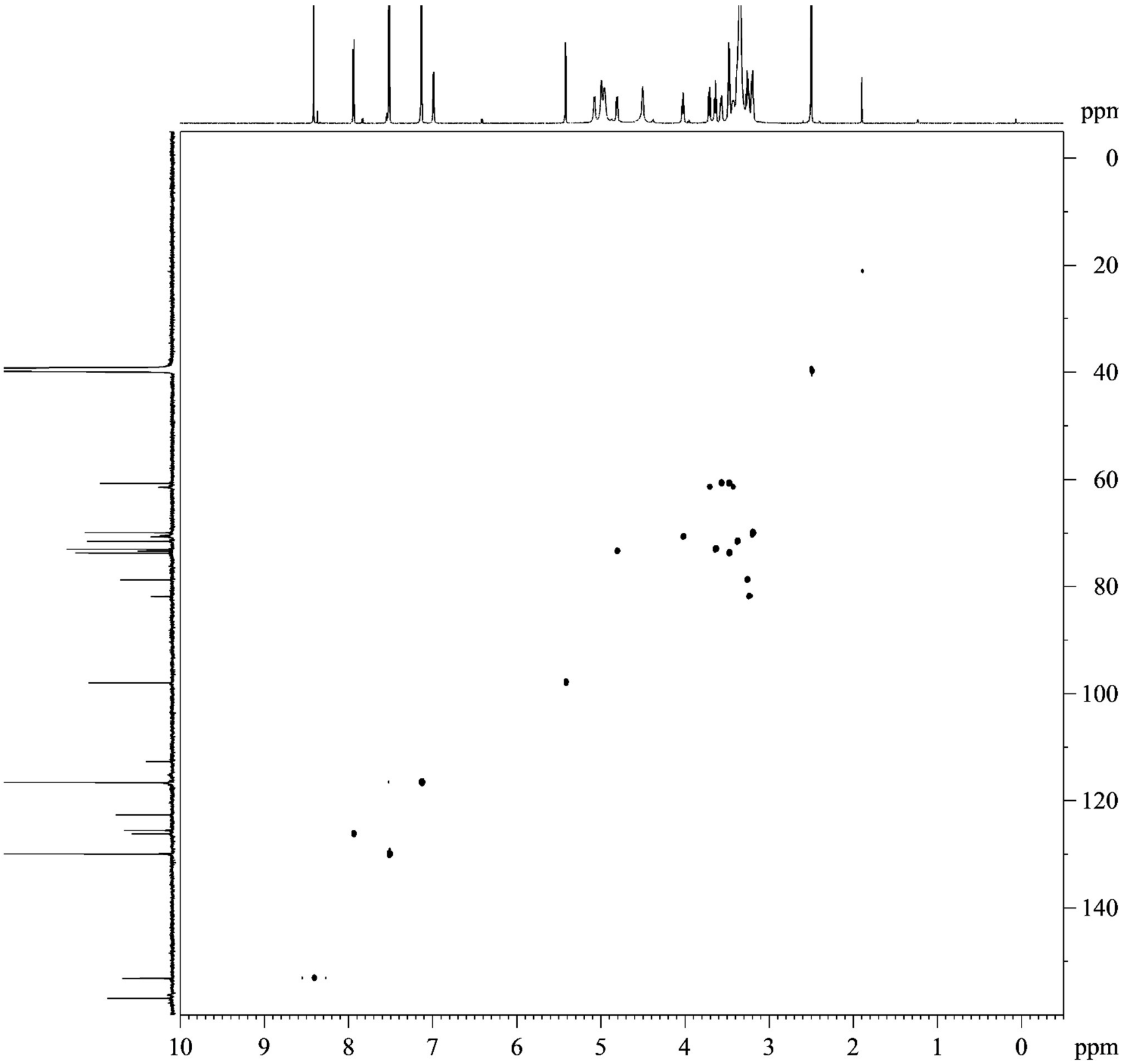

Figure S7. 2D NMR spectrum (^{1}H-^{13}C HSQC, 700 MHz, DMSO-d_6) of the puerarin-4'-*O*-β-glucoside (**1**).

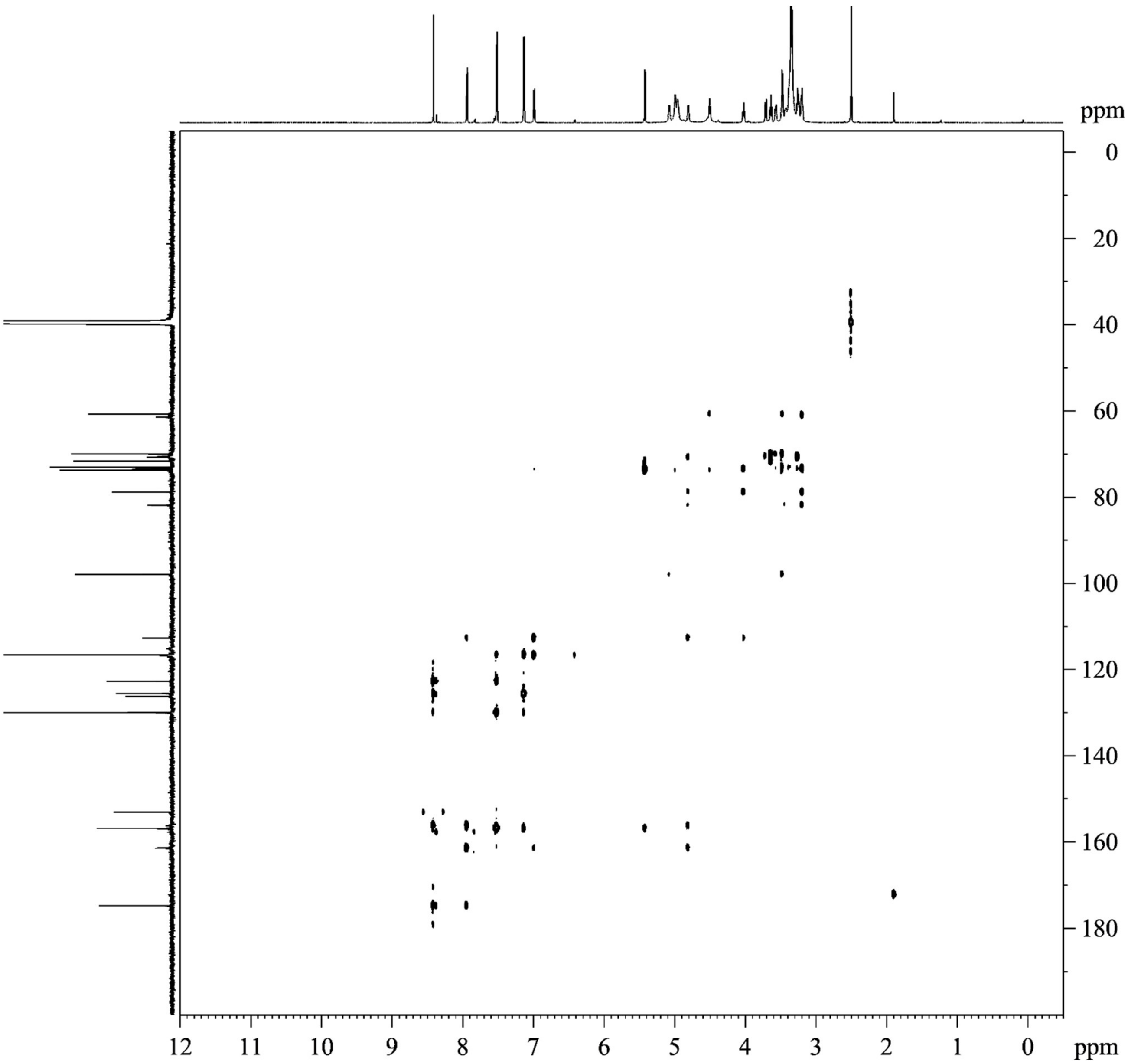

Figure S8. 2D NMR spectrum (^{1}H-^{13}C HMBC, 700 MHz, DMSO-d_6) of the puerarin-4'-*O*-β-glucoside (**1**).

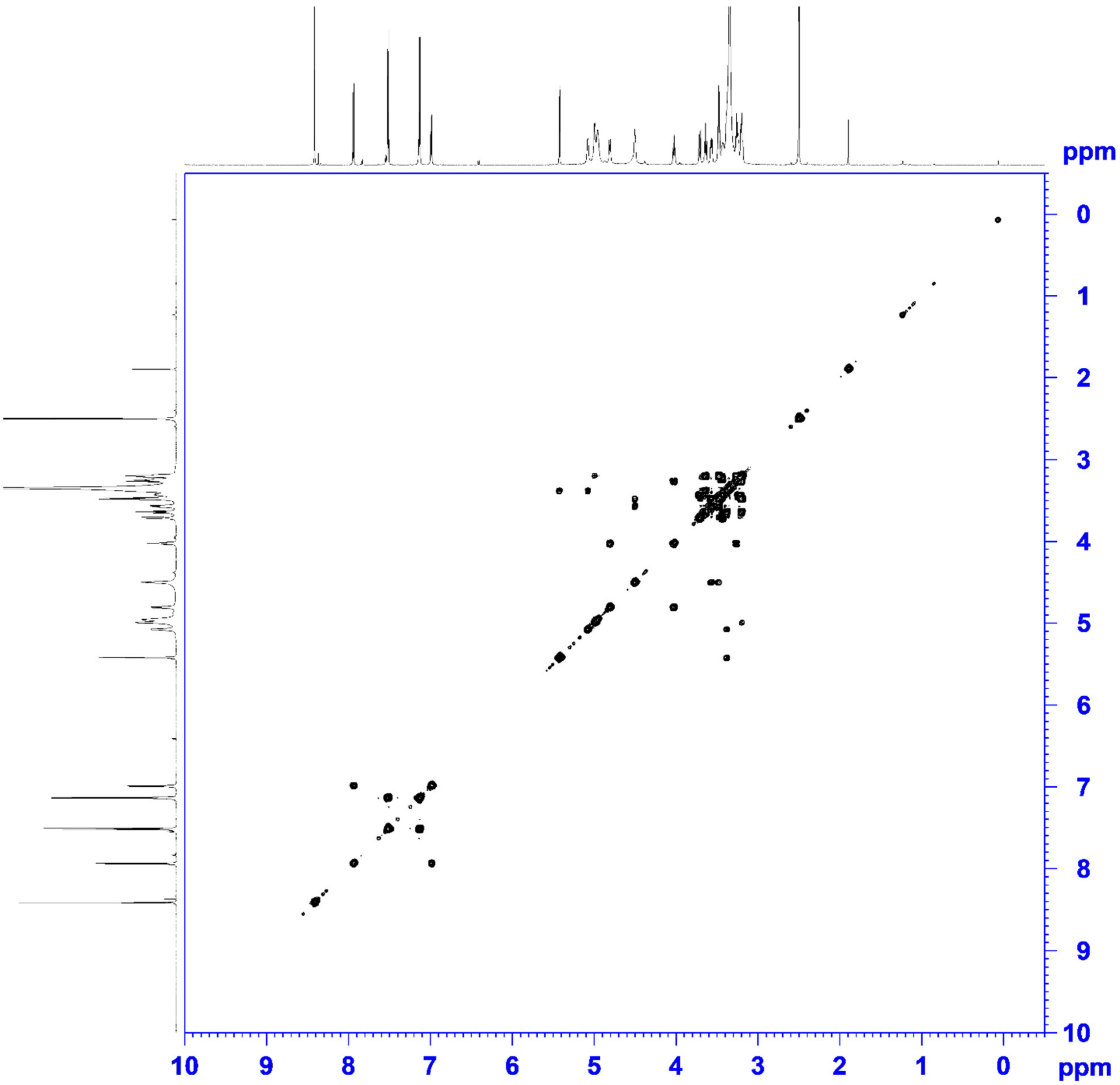

Figure S9. 2D NMR spectrum (^{1}H-^{1}H COSY, 700 MHz, DMSO-d_6) of the puerarin-4'-*O*-β-glucoside (**1**).

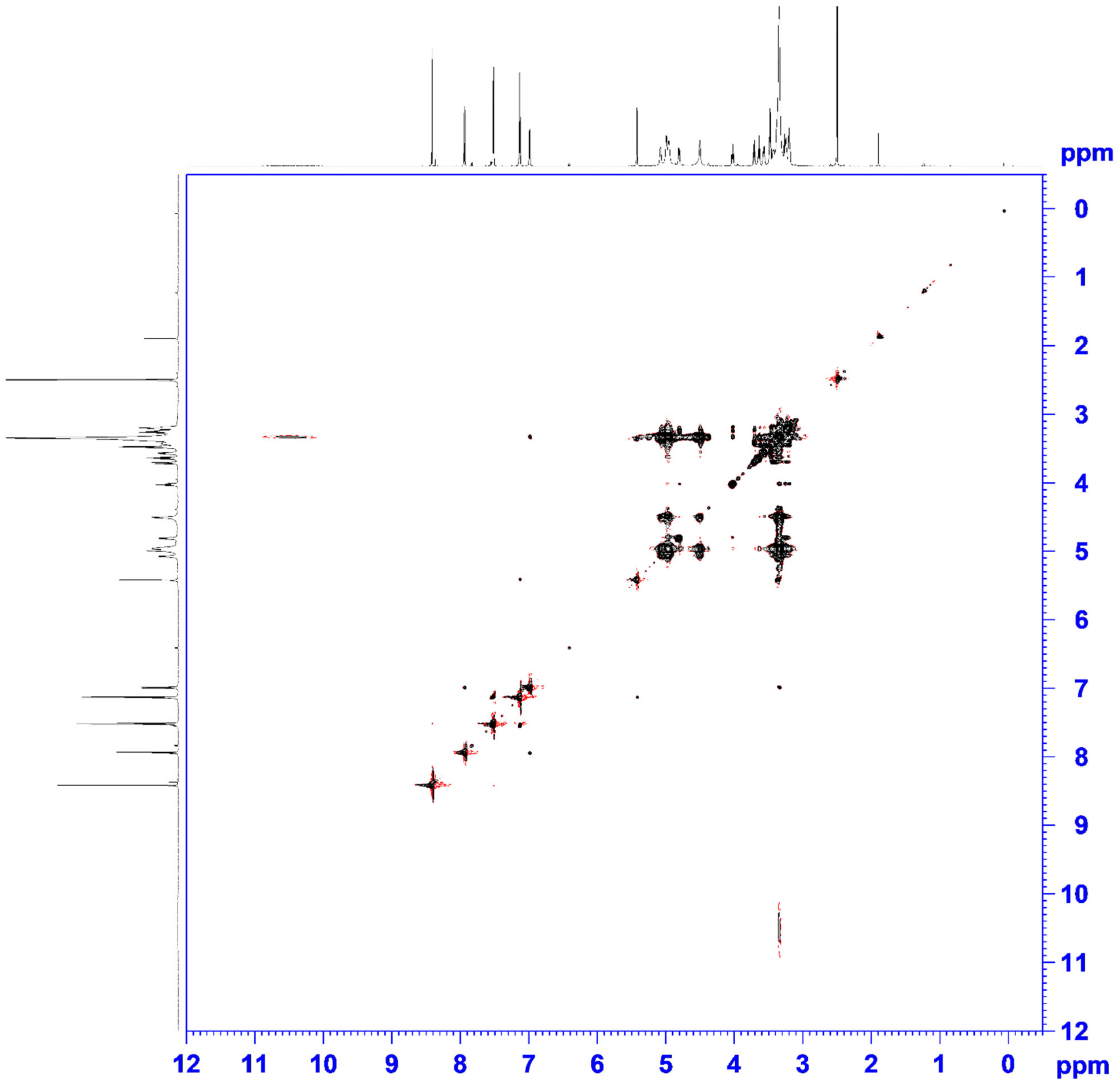

Figure S10. 2D NMR spectrum (^1H-^1H NOESY, 700 MHz, DMSO-d_6) of the puerarin-4'-*O*-β-glucoside (**1**).
